# Lipomas of the Hand: A Review and 13 Patient Case Series

**Published:** 2010-10-25

**Authors:** Menaka M. Nadar, Carlo R. Bartoli, Morton L. Kasdan

**Affiliations:** ^a^University of Louisville School of Medicine, Louisville, KY; ^b^Division of Plastic and Reconstructive Surgery, University of Louisville, Louisville, KY

## Abstract

**Objective:** In this article, the presentation, pathophysiology, diagnosis, treatment, and complications of lipomas of the hand are reviewed and evaluated. **Methods:** A thorough review of the literature is completed, and a series of 13 patients are summarized and briefly examined. **Results:** Lipomas may present as asymptomatic tumors or produce concerning signs and symptoms such as muscular atrophy and paralysis. Some lipomas may be identified by physical examination alone. However, magnetic resonance imaging best facilitates definitive diagnosis. **Conclusions:** In the absence of mechanical impairment or cosmetic concern, observation remains the clinical standard of care. When pain, compression neuropathy, disfigurement, or decreased function affect the patient, surgical resection is typically curative. Malignant transformation rarely occurs.

## BACKGROUND

Lipomas present as the most common tumor in the body.^1^ More common in obese individuals,^1^,^2^ these benign soft tissue neoplasms typically develop in the 5th to 7th decade of life.^1^,^3^ Lipomas are rarely found in children.^1^,^4^

**Figure 1:**
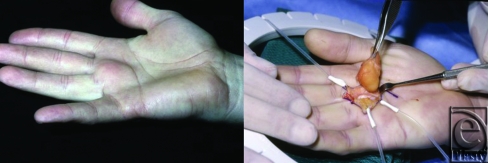
A 41-year-old man presented with a 2-year history of a right palmar mass between the long and ring fingers. Dissection revealed an encapsulated and multilocular lipoma.

Histologically, lipomas are nearly indistinguishable from normal adipose tissue.^1^ Composed primarily of mature adipocytes, these lesions are uniform in shape and size and well-circumscribed. A capsule typically surrounds a soft, yellow to orange lobular mass as shown in Figure 1. Although the histological appearance resembles mature adipose tissue, lipomas are not derived from mature adipocytes but rather from mesenchymal preadipocytes.^3^ Indeed, some variants of lipomas contain a heterogeneous mixture of other mesenchymally derived tissues. Related benign mesenchymomas include the following: fibrolipomas, which contain abundant fibrous tissue; angiolipomas, which are composed chiefly of mature adipocytes within extensive narrow vascular channels that contain fibrin microthrombi; chondrolipomas, which contain cartilaginous and lipomatous elements; myxolipomas, in which areas of mucoid mesenchymal tissues are intermixed with mature fat; myelolipomas, which contain adipocytes and hematopoietic tissue; and ossifying lipomas, which show osseous changes without a connection to bone.

The etiology of a lipoma is unknown. Multiple causative factors have been proposed that include genetic,^5^ traumatic,^6^^-^8 and metabolic^1^,^9^^-^13 triggers. The leading genetic theory for lipoma formation proposes that spontaneous karyotypic anomalies lead to chromosomal fusion products which promote proliferation of adipocytes.^5^ Although numerous other chromosomal aberrations have been described, lipomas are most commonly associated with translocations and rearrangements of the 12q13˜q15 chromosomal region.^5^,^14^ However, not all lipomatous neoplasms exhibit abnormal karyotypes.^15^ As an example, lipomas have been described in association with retinoblastoma gene mutations.^16^

In further support of a genetic origin, approximately 5% of patients with lipomas have multifocal lesions,^1^,^17^ and many of these individuals have a positive family history.^1^,^18^ Some families demonstrate an autosomal dominant mode of inheritance consistent with familial multiple lipomatosis.^19^ A simple dominant pattern has also been seen in Dercum's disease (adiposis dolorosa), which is typically observed in obese, postmenopausal women in whom numerous painful lipomas occur primarily around the hips and thighs.^20^ Furthermore, multiple lipomatous lesions are also components of several rare congenital syndromes that include Cowden's Syndrome,^21^ Bannayan-Zonana Syndrome,^22^ and Proteus Syndrome.^23^

Lipoma formation following physical trauma has been reported by multiple investigators. For decades, it was speculated that lipomatous tumors that arose after trauma were not actually a proliferation of adipocytes, but rather a herniation of preexisting adipose tissue through overlying fascia.^24^,^25^ These unencapsulated lesions were termed “pseudolipomas.” Later, a competing theory proposed that growth factors, cytokines, and other inflammatory mediators released following blunt trauma to soft tissue induced preadipocyte differentiation into mature adipocytes and formed a clinically apparent mass.^6^,^7^,^25^ More recently, it was suggested that fat necrosis and the extravasation of blood secondary to trauma stimulated preadipocyte differentiation.^8^,^26^ Interestingly, a spontaneous elevated partial thromboplastin time has been noted in 7 of 19 patients with posttraumatic lipomas.^25^ A novel, although unproven, theory is that microhemorrhage and focal release of cytokines secondary to a bleeding diathesis may trigger lipomatous growth.

Lipomas have been associated with numerous pathophysiological processes. Diabetes,^9^ hyperlipidemia,^1^ mitochondrial dysfunction,^10^ and endocrinopathies such as nodular goiter,^11^ multiple endocrine neoplasia type 1,^12^ and Cushing's syndrome^13^ have been noted. A case of lipoma development in a diabetic patient treated with chlorpropamide has also been reported.^27^ These findings suggest a role for metabolic dysfunction in the development of lipomatous neoplasms.

## LIPOMAS OF THE HAND

Lipomas were once believed to be rare in the upper extremity but are now considered common among soft tissue tumors of the hand.^28^ Most often found in subcutaneous fascia, lipomatous neoplasms occasionally occur in deeper layers. Development typically begins with an initial insidious growth period followed by a prolonged and latent maintenance state.^17^

Most often presenting as a solitary mass, hand lipomas are often asymptomatic and only come to clinical attention when they are of cosmetic concern or become large enough to create mechanical impairment. In Leffert's series of 141 lipomas of the upper extremity,^29^ 109 tumors were asymptomatic and excised solely for aesthetic reasons. Of the 32 symptomatic lesions, 26 caused pain or tenderness, and 6 produced paresthesias or sensory deficit secondary to nerve compression. Similar symptomatic presentations have been documented extensively in the literature. Lipomas that restricted range of motion and deformed the wrist or digits,^30^^-^2 decreased grip strength,^30^ or caused muscle paralysis,^33^ polyarthritis,^34^ trigger finger,^35^,^36^ dysesthesias,^35^ muscle atrophy,^33^,^35^ and nail plate dystrophy and thinning^37^ have been reported.

## DIAGNOSIS

**Figure 2:**
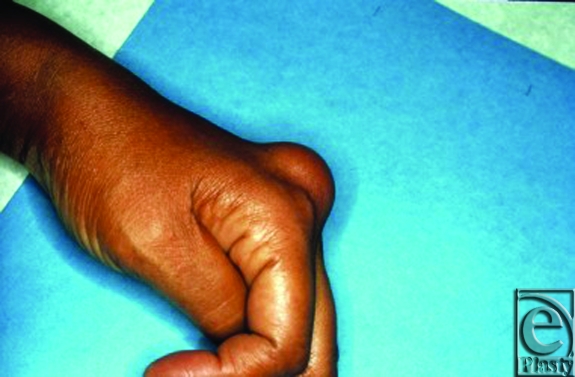
A 52-year-old woman presented with a subcutaneous lipoma on the dorsum of the left hand. The mass, located between the second and third metacarpals, was mobile with respect to the overlying skin but fixed to underlying structures.

History and physical examination are the foundation of diagnosis. As illustrated in Figure 2, lipomas most frequently present as a slowly enlarging, soft and mobile nontender mass. When subcutaneous, diagnosis can be made by a characteristic “doughy” feel on palpation. Application of an ice pack to the tumor to chill and harden the fat has also been used to aid in diagnosis.^38^

Occasionally, lipomas of the hand may be difficult to differentiate from ganglion cysts by palpation. Ganglion cysts allow passage of light on transillumination while lipomas do not.^39^ In patients presenting with symptoms of compression neuropathy, a positive Tinel's sign (distal paresthesia secondary to percussion over the tumor) may be helpful in confirming a lipoma as the source of nerve compression.^40^

When a subcutaneous tumor cannot be diagnosed by palpation, or when a lipoma develops within deeper structures where palpation is difficult, imaging may be necessary for diagnosis. On plain radiograph, lipomas appear as an area of characteristic radiolucency referred to as a “water-clear density.”^29^ Ultrasound examination demonstrates a homogeneous and circumscribed hyperechoic area.^41^,^42^ With computed tomography (CT), lipomas exhibit smooth edges with distinct margins,^43^ a uniform density comparable to normal fat,^44^,^45^ and do not enhance with intravenous contrast.^46^ Using magnetic resonance (MR) imaging, a homogeneous, high-intensity signal similar to subcutaneous fat may be observed.^31^,^47^

Both CT and MR imaging are reliable for localization,^48^ diagnosis,^45^,^48^,^49^ size estimation,^49^^-^51 as well as evaluation of bony involvement.^34^,^52^ Superior to plain-film radiographs, three-dimensional imaging allows for preoperative planning of approach, incision, and extent of dissection.^48^,^53^ Magnetic resonance imaging is preferable as it is both highly sensitive and specific for diagnosis.^54^ However, ultrasound may be used as a reasonable and cost-effective alternative if the suspicion for malignancy is low^55^ and the tumor is not contiguous with surrounding neurovascular or bony structures.^40^

## TREATMENT

**Figure 3:**
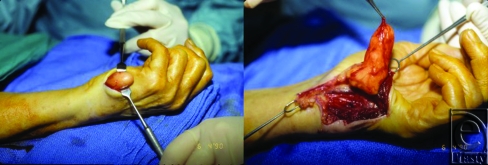
A 72-year-old woman presented with a lipoma of the left hypothenar eminence. The patient complained of swelling and tenderness over the ulnar aspect of the hand with numbness and tingling in the left ring and little fingers. Intraoperatively, the tumor was found to extend into Guyon's canal and could be traced to originate from the bifurcation of the motor and sensory branches of the ulnar nerve. Two months after resection, the patient was symptom-free with full use of the hand.

Small asymptomatic lesions that have been diagnosed by history and physical examination may be observed without intervention. However, surgical excision is indicated when pain, interference with hand function, compression neuropathy, or cosmetic concern are present.^29^,^30^ As demonstrated in Figure 3, the anatomical organization of the hand is complex, and a delicate dissection must be employed. The lipoma is usually surrounded by a thin, fibrous capsule, which may allow the mass to be shelled-out *in toto*. Marginal excision is appropriate and should result in complete resolution of symptoms over time^33^ that include restoration of sensation in cases of nerve compression.^3^,^51^ Rarely, in patients presenting with carpal tunnel syndrome, extensive nerve infiltration by a lipoma may not allow for tumor resection without causing permanent neurological damage. For such complex presentations, division of the flexor retinaculum without tumor excision is a good strategy for symptom relief.^56^

Alternative methods of tumor removal such as liposuction^57^ and endoscopically-assisted excision^58^ aim to minimize scarring. In addition, mesotherapy or intralesional phosphatidylcholine^59^ and deoxycholate^60^ injections have been used to shrink small lipomas. However, surgical resection or observation without intervention remain the standard of care.

## COMPLICATIONS

Lipomas rarely cause complications. However, patients with untreated compression syndromes may experience decreased neurological function and intractable neuropathic pain.^40^ In addition, there have been 2 unique reports of lipomas eroding into an adjacent metacarpal.^29^,^61^ Operative complications include neurovascular injury, hematoma, and hemorrhage.^62^ Division of nerves may produce enduring dysesthesias.^63^ Rarely, recurrence may be of concern and is typically associated with incomplete excision of deep, infiltrative lesions or lesions entangled within neurovascular structures.^64^ In patients with complicated anatomical infiltration or multilocular lesions, follow-up MR imaging to monitor for local recurrence is appropriate if symptoms develop.

## MALIGNANT VARIATION

Liposarcomas present as one of the most common soft tissue sarcomas of the body^1^ but are uncommon in the hand.^65^ Like lipomas, these tumors usually present as a small, slowly enlarging painless mass, although high-grade lesions may develop rapidly.^17^ Histologically, there is wide variation based on subtype and tumor grade, but all liposarcomas are defined by lipoblasts—malignant cells that recapitulate differentiating preadipocytes. The etiology of a liposarcoma is unknown, but most are thought to arise *de novo*.^1^,^17^ Reports of malignant transformation of lipomas are rare in the literature.^66^^-^8

CT may be used to visualize liposarcomas,^46^,^69^ but MR imaging is ideal.^54^,^70^ Findings on imaging are variable and dependent on degree of differentiation, but most show irregular or thickened septa^69^ and enhancement with gadolinium contrast.^71^,^72^ Features such as nodular, globular, or nonfatty areas as well as decreased fat composition also suggest malignancy.^49^

Treatment of liposarcomas requires wide local excision and in some cases may necessitate amputation.^65^ Occasionally, (neo)adjuvant chemotherapy or radiotherapy are administered,^73^^-^5 especially for high-grade lesions.^76^ The most common complications of liposarcomas are recurrence and metastasis.

## CLINICAL CASE SERIES

To illustrate the clinical presentation and management of lipomas of the hand, a series of 13 patients is briefly examined (Table 1). Seven male and 6 female patients presented at an average age of 57 years (range, 41-72). A variety of presentations were observed that included a single lesion in the left or right hand, dorsal or volar surface, and involvement of fingers or the wrist. The most common presenting symptoms were the description of a mass, swelling, pain, weakness, and decreased function. One patient complained of itching over the tumor site.

Surgical incision and approach were variable. Nearly all lipomas involved surrounding tissues such as nerves, vasculature, musculature, tendons, and skin, which illustrated the potential of these tumors to interfere with functions of the hand. As a result, most tumors required extensive dissection and lysis of adherent structures to ensure complete excision. Two of 13 cases required temporary silastic drains. The mean tumor size was 3.8 cm × 2.4 cm × 1.6 cm after fixation. Eleven of 13 patients returned for follow-up between 1 and 108 months. No recurrences were found.

## SUMMARY

This article outlines the current understanding of the pathophysiology, methods of diagnosis, treatment modalities, and complications of lipomatous tumors of the hand. Lipomas remain a common benign neoplasm of the hand. Simple subcutaneous lipomas may be diagnosed by history and physical examination alone. For more complicated tumors, MR imaging is most useful for diagnosis and preoperative planning. Most lipomas are treated with surgical excision with few complications or recurrence. Malignant degeneration is rare.

## Acknowledgment

The authors thank Jeremiah Redstone, MD for his assistance.

## Figures and Tables

**Table 1:** Lipomas of the Hand*

Patient No.	Age	Sex	Location	Presenting Symptoms	Tissue Involvement	Tumor Size	Operative Approach	Follow-up	Recurerence	Note
1	52	F	Dorsum of L hand, webspace of 2nd and 3rd metacarpals	N/A	Not fixed to overlying skin; fixed to extensor tendons of index and long fingers and dorsal interossei	N/A	Longitudinal incision, simple dissection, neurolysis, tenolysis, excision	46 months	No	—
2	50	M	R long finger, middle phalanx, dorsal radial aspect	N/A	Extensor tendon of long finger; dorsal branch of radial digital nerve	3.2 × 1.3 × 0.9 cm^3^	Transverse oblique fusiform incision, complex dissection, neurolysis, tenolysis, excision	108 months	No	—
3	41	M	R palm, between long and ring fingers	painless mass × 2 years	Radial digital nerve and artery of ring finger; ulnar digital nerve and artery of long finger	3 × 1.5 × 1.5 cm^3^	Modified Brunner incision, complex dissection, neurolysis, arteriolysis, excision	56 months	No	Figure 1
4	58	M	R index finger, proximal phalynx, radial aspect	painless mass × 2 weeks	Adherent to overlying skin; ulnar neurovascular bundle of index finger; dorsal surface of extensor tendon of index finger	2.5 × 2.2 × 1.1 cm^3^	Modified Brunner incision, complex dissection, arteriolysis, neurolysis, excision	1 month	No	—
5	63	M	R palm, thenar eminence	Swelling, aching, weakness of R thumb × 2 months	Radial neurovascular bundle of thumb	3 × 1.8 × 1.1 cm^3^	Brunner incision, complex dissection, arteriolysis, neurolysis, excision	N/A	N/A	—
6	72	F	L palm, hypothenar eminence	Swelling, tenderness over ulnar border of L hand × 14 months	Ulnar nerve at bifurcation of motor and sensory branches; Guyon's canal	4.5 × 2.5 × 1.2 cm^3^	Resection of previous scar along ulnar border, complex dissection, neurolysis, excision	26 months	No	History of previous attempted resection 1 month prior, **Figure 3**
7	N/A	F	Dorsum of R hand, webspace of 1st and 2nd metacarpals	Large mass	Princeps pollicis artery; ulnar neurovascular bundle of thumb; radial neurovascular bundle of index finger	6.5 × 4 × 4 cm^3^	Longitudinal incision, complex dissection, arteriolysis, neurolysis, excision, placement of silastic drains × 2	N/A	N/A	Closure with 2 silastic drains; **Figure 2**
8	58	F	L palm, webspace of 1st and 2nd metacarpals, extending into carpal tunnel	Swelling, itching × 4 months	Radial neurovascular bundle of index finger; noninvasive pressure on ulnar nerve	3.2 × 2.2 × 1 cm^3^	Longitudinal incision on dorsum of hand + classic carpal tunnel incision, complex dissection, neurolysis, tenolysis, excision, decompression L carpal tunnel	61 months	No	—
9	55	M	L forearm, deep to supinator	Unable to extend fingers or wrist × 3 months	Supinator; radial nerve	5 × 3.5 × 2.5 cm^3^	Transverse incision on dorsal wrist with exploration, curved incision on lateral aspect of elbow and forearm, complex dissection, neurolysis, excision	12 months	No	—
10	62	F	Dorsum of R hand	Enlarging mass × 30 months	Superficial fascia	2.2 × 1.1 × 0.6 cm^3^	Fusiform incision, simple dissection, excision	1 month	No	—
11	57	M	Distal volar aspect of L wrist	Progressively enlarging mass with discomfort × 2–3 years	Adherent to overlying skin and flexor retinaculum	2.6 × 1.9 × 0.7 cm^3^	Transverse incision, complex dissection, excision	1 month	No	—
12	71	F	L thumb, proximal phalynx, palmar aspect	Pain at base of left thumb and wrist	300° circumferential involvement of proximal phalynx; neurovascular bundle; flexor pollicis longus	4 × 3 × 3 cm^3^	Transverse incision, complex dissection, arteriolysis, neurolysis, tenolysis, excision	3 months	No	—
13	48	M	R palm	Hand cramps, mass × 72 months, enlarging × 3 months	Adductor pollicis; opponens pollicis; ulnar neurovascular bundle of thumb; radial neurovascular bundle of index finger	5.3 × 4 × 2 cm^3^	Counter incisions on anterior and posterior surfaces of webspace between 1st and 2nd metacarpals extending into thenar eminence, complex dissection, extensive neurolysis, excision, placement of silastic drains × 2	1 month	No	Closure with 2 silastic drains

*F, female; M, male; L, left; R, right; N/A, not availabale.
